# Specific Uptake and Genotoxicity Induced by Polystyrene Nanobeads with Distinct Surface Chemistry on Human Lung Epithelial Cells and Macrophages

**DOI:** 10.1371/journal.pone.0123297

**Published:** 2015-04-15

**Authors:** Vincent Paget, Samir Dekali, Thierry Kortulewski, Romain Grall, Christelle Gamez, Kelly Blazy, Olivier Aguerre-Chariol, Sylvie Chevillard, Anne Braun, Patrice Rat, Ghislaine Lacroix

**Affiliations:** 1 Institut National de l'Environnement Industriel et des Risques (INERIS), Unité de Toxicologie Expérimentale, Parc Technologique ALATA, BP2, Verneuil-en-Halatte, France; 2 Laboratoire de chimie et toxicologie analytique et cellulaire (C-TAC) / UMR CNRS 8638, Faculté de Pharmacie de Paris, Université Paris Descartes (PRES Sorbonne Paris Cité), Paris, France; 3 CEA, DSV, iRCM, Plateforme imagerie photonique, Fontenay-aux-Roses, France; 4 CEA, DSV, iRCM, Laboratoire Cancérologie Expérimentale (LCE), Fontenay-aux-Roses, France; Université de Technologie de Compiègne, FRANCE

## Abstract

Nanoparticle surface chemistry is known to play a crucial role in interactions with cells and their related cytotoxic effects. As inhalation is a major route of exposure to nanoparticles, we studied specific uptake and damages of well-characterized fluorescent 50 nm polystyrene (PS) nanobeads harboring different functionalized surfaces (non-functionalized, carboxylated and aminated) on pulmonary epithelial cells and macrophages (Calu-3 and THP-1 cell lines respectively). Cytotoxicity of in mass dye-labeled functionalized PS nanobeads was assessed by xCELLigence system and alamarBlue viability assay. Nanobeads-cells interactions were studied by video-microscopy, flow cytometry and also confocal microscopy. Finally ROS generation was assessed by glutathione depletion dosages and genotoxicity was assessed by γ-H2Ax foci detection, which is considered as the most sensitive technique for studying DNA double strand breaks. The uptake kinetic was different for each cell line. All nanobeads were partly adsorbed and internalized, then released by Calu-3 cells, while THP-1 macrophages quickly incorporated all nanobeads which were located in the cytoplasm rather than in the nuclei. In parallel, the genotoxicity study reported that only aminated nanobeads significantly increased DNA damages in association with a strong depletion of reduced glutathione in both cell lines. We showed that for similar nanoparticle concentrations and sizes, aminated polystyrene nanobeads were more cytotoxic and genotoxic than unmodified and carboxylated ones on both cell lines. Interestingly, aminated polystyrene nanobeads induced similar cytotoxic and genotoxic effects on Calu-3 epithelial cells and THP-1 macrophages, for all levels of intracellular nanoparticles tested. Our results strongly support the primordial role of nanoparticles surface chemistry on cellular uptake and related biological effects. Moreover our data clearly show that nanoparticle internalization and observed adverse effects are not necessarily associated.

## Introduction

The increasing production of engineered nanoparticles (NPs) for applications in a wide range of industrial processes and consumer products (such as drugs, food, cosmetics, surface coating, etc.) raise the problem of their effects on human health [1]. Manufactured NPs are defined as being in the nanoscale in any external dimensions [2] and can have multiple chemical surface functionalizations depending on their application. Inhalation is a major route for NPs exposure and, in contrast to large particles (normally cleared by the upper airways), NPs can be deposited by diffusion mechanisms in all structures along the respiratory tract, from the head airways to the alveoli, entering into cells easily and possibly inducing cytotoxic effects [3–7]. Although airways and alveoli have their own specificities and functions, they exhibit the same basic structural elements: i) the liquid liner layer, ii) the mobile cells (resident airway or alveolar macrophages), iii) the epithelium with adherent and tight junctions between cells, and iv) the sub epithelial connective tissue with blood and lymphatic vessels and other immune cells [7]. Macrophages and epithelial cells are thus the first target for inhaled NPs. A major function of macrophages is to remove particles and opsonized NPs that reach deeper airways [8].

Due to their tights junctions, epithelial cells form a physical barrier in airways and alveoli against inhaled particles. However, it has been shown that they are also able to internalize NPs [6]. Recent studies have reported that surface chemistry could strongly impact NPs interactions with pulmonary cells [9,10]. Lunov *et al*. have recently shown that primary human macrophages could internalize carboxylated polystyrene (PS) nanobeads in HBSS (Hank’s Balanced Salt Solution) *via* clathrin- and dynamin-dependent endocytosis, while macropinocytosis appeared to play a predominant role after exposure to aminated PS nanobeads in HBSS [11]. In biological media, proteins can rapidly adsorb on NPs surface forming the “protein corona” but this phenomenon is strongly dependent on NPs surface chemistry and could influence NPs internalization by cells. Indeed, Lunov *et al*. previously showed that internalization of PS NPs did not involve phagocytosis by human macrophages probably because of a lack of opsonizing plasma proteins. Fröhlich *et al*. showed a reduced cellular uptake by the endothelial EAhy926 cell line due to the presence of these proteins [11,12]. The NPs uptake by macrophages or epithelial cells plays a central role in biological responses such as direct or indirect production of reactive oxygen species (ROS). Mechanisms of cell damages such as inflammation, genotoxicity and apoptosis caused by NPs are often explained by the production of ROS [13]. Shukla *et al*. recently showed on the human epidermal A431 cell line that internalized TiO_2_ NPs induced a significant reduction of glutathione and ROS generation in association with oxidative DNA damage and micronucleus formation [14]. Moreover, other authors demonstrated that indirect cytotoxic effects could also occur *via* ROS generated by primary apoptotic intestinal Caco-2 cell line, which then induced apoptosis in neighboring cells [15].

Even though several studies have reported the ability of NPs to induce DNA damages [16,17], only few studies have focused on genotoxic effects related to NPs surface chemistry [17–20]. Moreover, to our knowledge, there is no published data on potential genotoxic effects of polystyrene NPs related to their surface chemistry. These NPs are widely used in nanotoxicology for studying cellular uptake because they are easily traceable by fluorescence, often synthesized in research laboratories [11,21], but also commercially available with reproducible sizes and surface chemistry and exhibiting extremely slow degradation. Moreover, polystyrene nanoparticles are commonly found in spray and exterior paints and are also used in electronics and diagnostics processes.

Here, the goal was to investigate specific uptake and links with the cytotoxic effects (oxidative stress and genotoxicity) induced by polystyrene nanobeads with distinct surface chemistry. As macrophages and epithelial cells are the first target of inhaled pollutants throughout the respiratory tract, we used THP-1 differentiated cells as a model for lung macrophages [11,22–24] and Calu-3 cells as a model for lung epithelium junctions [25–28] Human cell lines easily accessible were chosen in order to ease the implementation of methods. Moreover, we investigated three sets of perfectly characterized PS nanobeads with different surface chemistries, non-functionalized (PS-NF), carboxylated (PS-COOH) and aminated (PS-NH_2_), on each cell line, using PS nanobeads concentrations ranging from 1 to 100 μg/ml corresponding to 0.3 to 32.3 μg/cm^2^, respectively. According to Paur *et al*. [29] the lowest dose (0.32 μg/cm^2^) corresponds to the dose that would be received in the lung of a person exposed to 5 mg/m^3^ during 3 days (assuming no clearance and a tissue deposition efficiency of 30%).

On one hand, we assessed the effects of PS NPs on cell viability, oxidative stress and genotoxicity. To perform this work, we used innovative approaches to monitor real time cell viability and morphology by impedance measurements using the xCELLigence system. Conventional alamarBlue viability assay was used to corroborate obtained results. We also measured intracellular glutathione known to play a critical role in the cellular defense against oxidative stress agents [30]. To quantify DNA double strand breaks the γ-H2Ax foci detection method was used as it has been previously described as a highly sensitive assay [21,31] and a good predictor of *in vivo* genotoxicity [32].

On the other hand, we explored the correlation between the uptake of these different PS nanobeads and cellular damages. Cellular uptake was analyzed by video-confocal microscopy (real-time monitoring), flow cytometry (quantitative approach of NPs-cells interactions) and confocal fluorescence microscopy (cellular localization of NPs).

## Results

### Physico-chemical characterization of polystyrene nano-beads in relevant biological media

**Fig 1:**
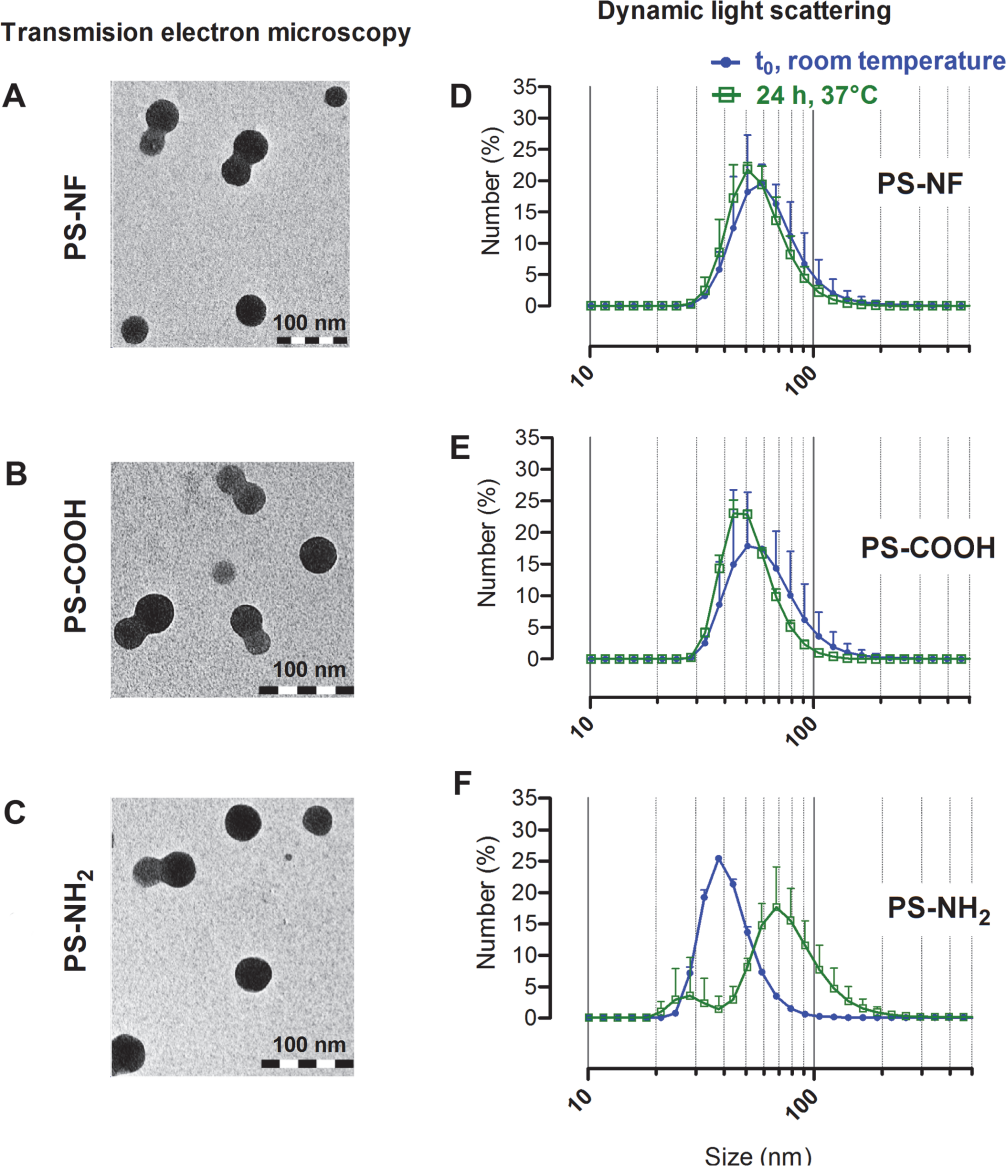
Transmission electron microscopy images (TEM) and size distributions of PS nano-beads. NPs were suspended in RPMI 1640 cell culture media supplemented with 5% (v/v) FBS and 1% (v/v) penicillin/streptomycin. TEM images (column 1) show non-functionalized (A and D), carboxylated (B and E) and aminated (C and F) polystyrene nanobeads (scale bars = 100 nm). Size distributions (column 2) of each nanobeads were determined by dynamic light scattering at t_0_ at room temperature (blue curves) and after 24h in an incubator at 37°C (green curves).

In this study, we used 3 different PS nanobeads with different surface chemistries: non-functionalized (PS-NF), carboxylated (PS-COOH) and aminated (PS-NH_2_) nanobeads. PS-NF and PS-COOH nanobeads were indirectly sonicated with a cup-horn at room temperature, while PS-NH_2_ nanobeads were just vortexed before use. Indeed, we observed that sonication induced PS-NH_2_ nanobeads aggregates (S1 Fig). Transmission Electron Microscopy (TEM) analysis showed that all nanobeads were mainly individualized after dispersion in complete cell culture medium with few small aggregates of two to three NPs (Fig 1, column 1). Moreover, no significant chemical contamination of nanobeads was observed using the energy-dispersive X-ray microanalyser (data not shown). Dynamic Light Scattering (DLS) measurements confirmed that PS nanobeads were mono-dispersed with low polydispersity index (PDI < 0.08) after dispersion in water (Fig 1, column 2 and Table 1). In complete cell culture medium, PDI were higher, suggesting slight PS nanobeads agglomeration for PS-NF and PS-COOH. No marked change was observed in particle size distribution when measuring after 24 h incubation at 37°C (Fig 1D and 1E), except for PS-NH_2_ nanobeads which exhibited a significant increase of the PDI and the formation of small aggregates mainly < 100 nm (Fig 1F and Table 1). Despite their different surface functionalization, PS-NF and PS-COOH nanobeads had a negative potential in water and in complete culture medium (Table 1). In contrast, PS-NH_2_ nanobeads had a positive zeta potential in water while it became close to zero in complete culture medium (Table 1).

**Table 1:** Zeta potential and polydispersity indexes of PS nanobeads.

Nanobeads	Zeta-potential (mV)	Polydispersity indexes	
		*t_0_, room temperature*	*24 h*, *37°C*
**PS-NF^(^^A^^)^**	- 60.4 ± 1.6	0.04 ± 0.01	Not performed
**PS-COOH^(^^A^^)^**	- 61.3 ± 1.1	0.05 ± 0.01	Not performed
**PS-NH_2_^(^^A^^)^**	40.9 ± 0.8	0.06 ± 0.01	Not performed
**PS-NF^(^^B^^)^**	-13.7 ± 0.8	0.20 ± 0.02	0.15 ± 0.06
**PS-COOH^(^^B^^)^**	-14 ± 0.3	0.14 ± 0.05	0.10 ± 0.02
**PS-NH_2_^(^^B^^)^**	-5.06 ± 0.5	0.22 ± 0.09	0.59 ± 0.02

^(A)^ in water

^(B)^ in complete culture medium

Measurements were performed on PS nanobeads suspended in water (A) and on PS nanobeads suspended in RPMI 1640 supplemented with 5% (v/v) FBS (B). Data represent the mean ± SD of three independent experiments.

### xCELLigence (real-time follow-up) and cytotoxicity

**Fig 2:**
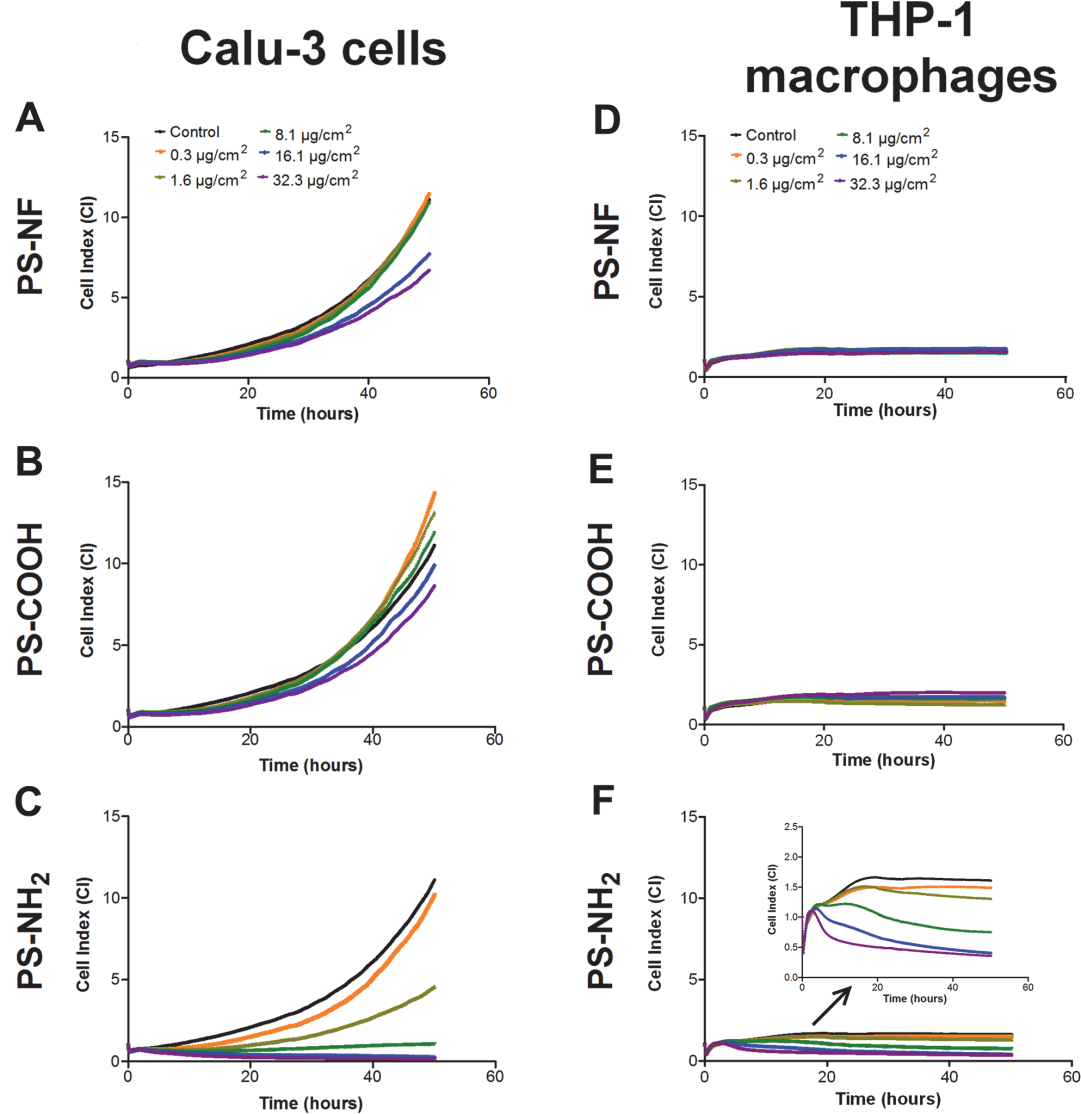
Cell index real-time monitoring of Calu-3 epithelial cells and THP-1 macrophages exposed to PS nanobeads. Cell index real-time monitoring of Calu-3 epithelial cells and THP-1 macrophages are reported on column 1 and 2 respectively. One representative experiment among three independents experiments were carried out for 48h and cell indexes were normalized at time 0 to ensure non inter-wells variability prior to the addition of nanoparticles. Calu-3 epithelial cells and THP-1 differentiated macrophages were exposed 48 h to the three kinds of PS nanobeads, PS-NF (A and D), PS-COOH (B and E) and PS-NH_2_ (C and F).

Real-time monitoring of living cells using xCELLigence technology is a useful high throughput screening method allowing to detect transient responses and remarkable time points after exposure. Calu-3 cells were seeded on E-plates 48 h before exposure, while THP-1 monocytes were seeded and induced to differentiate with PMA 24 h before exposure. Then, cell index (CI) was normalized (time 0) just before addition of nanobeads to the cell culture. After 24 h of PMA incubation, we observed that cell index (CI) values of non NPs-exposed differentiated THP-1 macrophages remained constant until the end of the experiment, testifying of adherence and absence of cell proliferation (S2 Fig). THP-1 monocytes without PMA treatment were also used as controls and were monitored throughout the experiment (S2 Fig). Real-time follow-up of Calu-3 cells and THP-1 macrophages is showed on Fig 2. PS-NF and PS-COOH nanobeads did not induce significant response compared to non-exposed cells up to 48 h (Fig 2A and 2B for Calu-3 cells, and Fig 2D and 2E for THP-1 macrophages), excepted for Calu-3 cells exposed 48 h to PS-NF nanobeads where CI variations were observed compared to control (Fig 2A). In parallel, the alamarBlue viability assay did not show any significant difference up to 48 h between control and PS-NF or PS-COOH exposed Calu-3 cells (S3A and S3B Fig) and THP-1 macrophages (S3D and S3E Fig). Therefore, alamarBlue results clearly confirmed that the CI variations observed at 48 h for Calu-3 cells exposed to PS-NF were attributable to cell morphological changes rather than to cell mortality.

In contrast with PS-NF and PS-COOH nanobeads, PS-NH_2_ nanobeads induced dose–dependent CI decreases for all concentrations above 1.6 μg/cm^2^ compared to the control for Calu-3 cells (Fig 2C) and THP-1 macrophages (Fig 2F). These results were confirmed by the alamarBlue assay, showing a significant decrease of cell viability starting 4 h after exposure for concentrations above 1.6 μg/cm^2^ for Calu-3 cells (S3C Fig), and 24 h after exposure to 32.3 μg/cm^2^ for THP-1 macrophages (S3F Fig).

### PS nanobeads uptake

**Fig 3:**
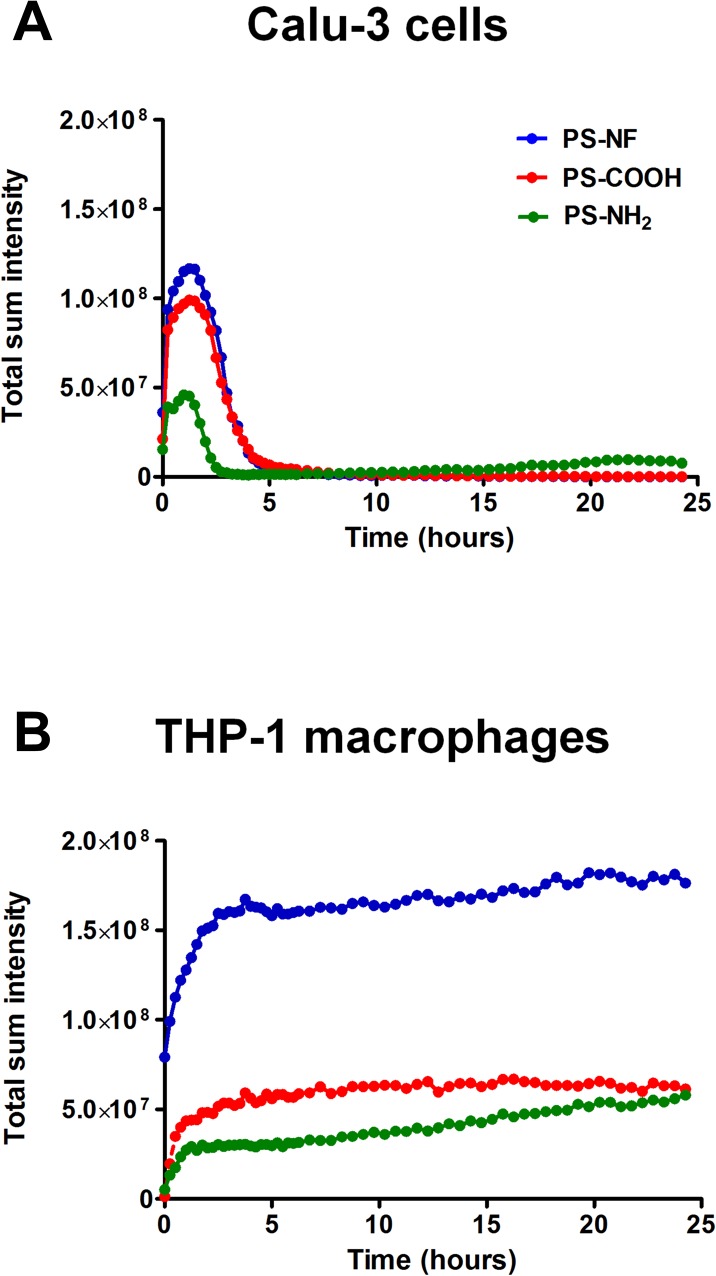
Fluorescence follow-up of Calu-3 cells and THP-1 macrophages exposed to PS nanobeads. Total sum intensity of fluorescence performed by 24 h real-time video-microscopy follow-up on Calu-3 cells (A) and THP-1 macrophages (B) exposed to PS-NF (blue curve), PS-COOH (red curve) and PS-NH_2_ (green curve) nanobeads.

**Fig 4:**
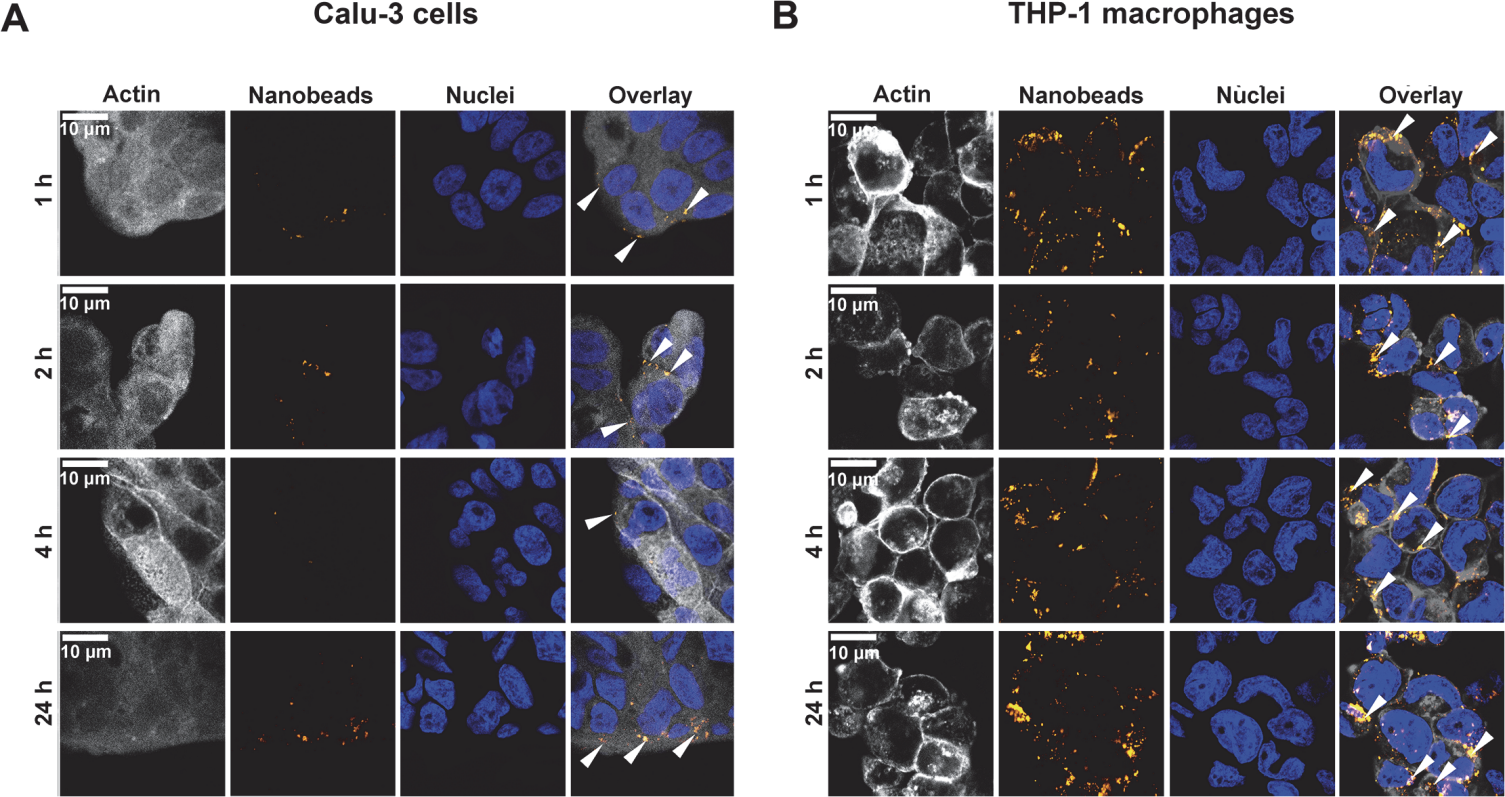
Confocal microscopy images of Calu-3 cells and THP-1 macrophages exposed to PS-NH_2_ nanobeads. Confocal microscopy images of Calu-3 cells are reported on (A) and in (B) for THP-1 macrophages. For both cell types, times of exposure are as follows: 1 (line 1), 2 (line 2), 4 (line 3) and 24 h (line 4). Cytoplasm staining (actin) is represented on column 1, nanobeads on column 2, DNA staining on column 3 and column 4 represents overlay of the three stainings. White arrows indicate nanobeads location on overlay images (column 4). White bars correspond to 10 μm.

We first monitored PS nanobeads cellular-uptake by video-microscopy. Our results clearly indicate that Calu-3 cells and THP-1 macrophages presented distinct uptake profiles after an exposure to 8.1 μg/cm^2^ of nanobeads (S4 Fig). PS nanobeads appeared to be poorly incorporated in Calu-3 epithelial cells (S4A Fig) while they were rapidly internalized in the cytoplasm of THP-1 cells (S4B Fig). To quantify nanobeads cellular-uptake, we summed in projection the pixels intensity of each acquisition throughout the experiment using NIS Elements software (Tokyo, Japan) (Fig 3). For Calu-3 cells (Fig 3A) a continuous and exponential increase of the total sum of pixel intensity was observed until 2 h exposure, followed by a rapid decrease between 2 and 4 h back to the basal level. For THP-1 macrophages, data indicated that all nanobeads were rapidly internalized after exposure with a peak of intensity 2 h after exposure, followed by a slight increase of total intensities for PS-NF and PS-COOH nanobeads (Fig 3B). After 2 h exposure, the slope of the curve was higher for PS-NH_2_ nanobeads, indicating a continuous accumulation of nanobeads into THP-1 macrophages (Fig 3B). These results were confirmed by flow cytometry analysis (S5 Fig). From 4 to 24 h in Calu-3 cells, a decrease of PS-NF and PS-COOH nanobeads-cells interactions was observed, while these interactions increased for PS-NH_2_ nanobeads (S5A Fig). In contrast, for the three nanobeads, a continuous higher number of PS nanobeads/positive cells was observed throughout the experiment for THP-1 macrophages (S5B Fig). In order to precise nanobeads cellular localization (external and/or within cells), we also performed confocal microscopy experiments, especially to verify the particular profile of PS-NH_2_ nanobeads on Calu-3 cells. Fluorescence pictures of cells exposed to PS-NH_2_ nanobeads are represented on Fig 4A for Calu-3 cells and Fig 4B for THP-1 macrophages, respectively. These results clearly confirmed that nanobeads were mainly located around Calu-3 cell islets 4 h post exposure and within the cells at 1, 2 and 24 h (Fig 4A). The same analyses on THP-1 macrophages showed that PS-NH_2_ nanobeads were located in the cytoplasm from 1 to 24 h of exposure (Fig 4B). Similar experiments were performed for PS-NF and PS-COOH nanobeads (data not shown) and showed that until 4 h nanobeads were external to Calu-3 cells, and within the cytoplasm of THP-1 (data not shown).

### Genotoxicity

Genotoxicity results measured by analyzing γ-H2Ax foci on Calu-3 cells and THP-1 macrophages, are reported in Table 2. Data indicated that PS-NF nanobeads did not significantly induce genotoxicity in both cell lines, excepted after exposure to 8.1 μg/cm^2^ PS-NF at 1 h for THP-1 macrophages for which a faint but significant increase (*, *p* < 0.05) of γ-H2Ax foci was observed compared to control cells. After PS-NF nanobeads exposure, although no significant increase of the γ-H2Ax foci was detected compared to the control, a downward trend of median values was observed at 1 and 2 h for both cell lines (Table 2). PS-COOH nanobeads did not induce genotoxicity in Calu-3 cells (Table 2), while they increased slightly but significantly the number of γ-H2Ax foci in THP-1 macrophages, between 1 h and 24 h (Table 2). PS-NH_2_ nanobeads induced genotoxicity in both cell lines as seen by the distributions of γ-H2Ax foci that were significantly different from that of control, even for the lowest concentration (0.3 μg/cm^2^) (***, *p* < 0.001) (Table 2).

**Table 2:** Genotoxicity of PS nanobeads on Calu-3 cells and THP-1 macrophages.

			1 h			2 h			4 h			24 h		
			Control	0.3 μg/cm^2^	8.1 μg/cm^2^	Control	0.3 μg/cm^2^	8.1 μg/cm^2^	Control	0.3 μg/cm^2^	8.1 μg/cm^2^	Control	0.3 μg/cm^2^	8.1 μg/cm^2^
**Calu-3 cells**	**PS-NF**	Min.	0	0	0	0	0	0	0	0	0	0	0	0
		25th perc.	1	1	1	1	1	1	1	1	1	1	1	1
		**Median**	**3**	**4**	**4**	**3**	**4**	**4**	**3**	**3**	**3**	**3**	**4**	**3**
		75th perc.	9	13	13.5	9	10	11	9	9	11	9	9	11
		Max.	53	59	73	78	71	75	55	80	79	72	81	73
		SA	-	**NS**	**NS**	**-**	**NS**	**NS**	**-**	**NS**	**NS**	**-**	**NS**	**NS**
	**PS-COOH**	Min.	0	0	0	0	0	0	0	0	0	0	0	0
		25th perc.	1	1	1	1	1	1	1	1	1	1	1	1
		**Median**	**3**	**3**	**4**	**3**	**3.5**	**3**	**3**	**4**	**3**	**3**	**4**	**3**
		75th perc.	9	10	13	9	11	14	9	9	10	9	9	9
		Max.	53	83	89	78	89	85	55	78	78	72	73	85
		SA	-	**NS**	**NS**	-	**NS**	**NS**	-	**NS**	**NS**	-	**NS**	**NS**
	**PS-NH**_**2**_	Min.	0	0	0	0	0	0	0	0	0	0	0	0
		25th perc.	1	3	3	1	4	4	1	3	4	1	3	4
		**Median**	**3**	**9**	**10**	**3**	**10**	**10**	**3**	**8**	**9**	**3**	**7**	**8**
		75th perc.	9	26	26	9	19.3	25	9	23	21	9	21	18.3
		Max.	53	92	115	78	124	148	55	110	119	72	106	96
		SA	-	***	***	-	***	***	-	***	***	-	***	***
**THP-1 macrophages**	**PS-NF**	Min.	0	0	0	0	0	0	0	0	0	0	0	0
		25th perc.	1	3	3	1	2.5	3	1	3	3	1	3	3
		**Median**	**7**	**10**	**11**	**7**	**9**	**9**	**7**	**9**	**9**	**7**	**8**	**9**
		75th perc.	20	20	21	20	21	22	19.8	21	20	19.3	20	20.3
		Max.	86	82	85	75	83	87	81	80	89	72	87	87
		SA	-	**NS**	*	-	**NS**	**NS**	-	**NS**	**NS**	-	**NS**	**NS**
	**PS-COOH**	Min.	0	0	0	0	0	0	0	0	0	0	0	0
		25th perc.	1	2.5	4	1	3	3	1	3	3	1	1	3
		**Median**	**7**	**10**	**11**	**7**	**9**	**11**	**7**	**9**	**10**	**7**	**8.5**	**10**
		75th perc.	20	22	21.3	20	22	21.8	19.8	20	21.8	19.3	81	22
		Max.	86	89	81	75	73	87	81	74	89	72	21	77
		SA	-	*	**	-	**NS**	*	-	**NS**	*	-	**NS**	*
	**PS-NH**_**2**_	Min.	0	0	0	0	0	0	0	0	0	0	0	0
		25th perc.	1	7	7	1	9	8.3	1	6	8	1	7	7
		**Median**	**7**	**15**	**16**	**7**	**16**	**16**	**7**	**16**	**16**	**7**	**15**	**15**
		75th perc.	20	26	27	20	27.5	28.8	19.8	29	28	19.3	27	28.3
		Max.	86	98	103	75	94	97	81	89	97	72	95	99
		SA	-	***	***	-	***	***	-	***	***	-	***	***

PS nanobeads genotoxicity measured by γ-H2Ax-foci counts on Calu-3 cells and THP-1 macrophages. Counts were performed on at least 200 cells per condition and results are depicted as box plot distribution values [minimum (min), maximum (max), median, 25^th^ and 75^th^ percentiles (25^th^ and 75^th^ perc.)] of the foci number obtained in each tested condition. A Wilcoxon rank test (comparisons versus control cells not exposed to NPs) was performed (* = *p*<0.05; ** = *p*<0.01; *** = *p*<0.001).

### Oxydative stress: GSH depletion

Reduced GSH was measured in Calu-3 cells and THP-1 macrophages exposed to PS nanobeads using the monochlorobimane (mBCI) assay. PS-NF nanobeads exposure (for concentrations from 8.1 to 32.3 μg/cm^2^) led to a transient decrease of GSH after 4 h exposure for Calu-3 cells and after 1 h exposure for THP-1 macrophages (S6A and S6D Fig). Furthermore, significant decreases were observed in response to the highest concentrations of PS-COOH nanobeads (16.1 and 32.3 μg/cm^2^) after 4 h exposure for Calu-3 cells (S6B Fig), while this depletion was observed after 1 and/or 2 h of exposure for THP-1 macrophages (S6E Fig). Significant decreases were also observed for Calu-3 cells and THP-1 macrophages from 1 h to 24 h (S6C and S6F Fig, respectively) in response to PS-NH_2_ nanobeads exposure (for concentrations from 8.1 to 32.3 μg/cm^2^).

## Discussion

The aim of this work was to correlate, jointly to nanobeads internalization, the impact of NPs surface chemistry on cell response by studying cytotoxicity and genotoxicity. To be sure to ascribe any observed effect to only the surface chemistry of PS nanobeads, it was a prerequisite to treat cells with characterized and mono-dispersed nanobeads suspension. For this reason we performed DLS measurements and TEM analysis to control nanobeads dispersions. No marked changes of nanobeads suspension particle size were observed after 24 h at 37°C except for PS-NH_2_ for which a significant increase of the PDI and the formation of small aggregates < 100 nm were observed (Fig 1). Moreover, PS nanobeads suspensions were stable and remained well dispersed at least 48 h after their preparation, ensuring a high reproducibility of biological experiments during this period (data not shown). The three sets of perfectly characterized PS nanobeads were investigated in two human cell lines: Calu-3 epithelial cells and THP-1 differentiated macrophages using PS nanobeads concentrations (from 1 to 100 μg/ml, corresponding to 0.3 and 32.3 μg/cm^2^, respectively) in the same range of doses used in other published studies [33,34].

Using the xCELLigence system, we performed a first screening of PS nanobeads cellular effects. This method based on impedance measurement upon adherent cells allows real-time recording of signal. It allowed us to control adhesion of THP-1 monocytes becoming THP-1 differentiated macrophages after 24 h of incubation with PMA (S2 Fig), a protocol previously described in the literature [35]. One of the advantages of this system is to avoid interferences with NPs, a key point since a lot of studies already reported such NPs interferences with conventional colorimetric or fluorimetric toxicological assays [36–40]. Calu-3 cells and THP-1 macrophages real-time monitoring showed similar cell responses after PS nanobeads exposure. PS-NF and PS–COOH nanobeads did not induce significant responses compared to non-exposed cells while strong effects were observed on cell viability for PS-NH_2_ nanobeads (Fig 2 and S3 Fig), indicating a key role of the surface chemistry. Indeed PS-NH_2_ nanobeads induced a dose-dependent CI decreases for all concentrations above 1.6 μg/cm^2^ compared to the control for Calu-3 cells (Fig 2C) and THP-1 macrophages (Fig 2F). All xCELLigence results were confirmed by the alamarBlue assay (S3 Fig). Consequently, the decrease of CI values was mainly due to cell mortality rather than cell morphological changes. These cytotoxicity data are consistent with previously published data on human cell lines, reporting that PS-NH_2_ nanobeads were more cytotoxic than PS-NF or PS-COOH nanobeads [33,34,41–46]. Si-NH_2_ NPs have also been reported to induce more potent cytotoxicity and ROS generation on murine macrophages (NR8383 cells) compared to Si-COOH NPs [47,48]. Finally, we showed that PS-NH_2_ nanobeads induced cytotoxicity after exposure of Calu-3 cells and THP-1 macrophages in dose ranges similar to ones previously published (IC50 between 31 and 75 μg/mL, depending on the cell lines) [33,34].

As several studies have already demonstrated that nanobeads can induce bystander effects, without being internalized by cells [15,49], we then studied nanobeads uptake to be able to further address the issue on direct or indirect cellular effects at non-cytotoxic doses. We successively used three complementary detection approaches commonly used in research laboratories: i) a real-time monitoring of NPs uptake by video confocal microscopy, ii) a semi-quantitative detection of interactions between nanobeads and external cellular membrane using flow cytometry, iii) a qualitative NPs detection by confocal microscopy acquisitions to precisely locate NPs within and/or around the cells. Video-microscopy monitoring for 24 h after PS nanobeads exposure showed distinct time kinetics of NPs accumulations for Calu-3 cells (Fig 3A and S4A Fig) and THP-1 macrophages (Fig 3B and S4B Fig), possibly related to different uptake mechanisms between epithelial cells [50] and macrophages [11,51]. However, due to the low resolution (1 pixel representing 640 nm), only NPs accumulation was informative. Moreover, quantification of intensity could not be directly compared between all conditions because: i) for a same quantity of different PS nanobeads, fluorescence intensities were not exactly the same ii) in each field of acquisition, the number of exposed cells was not exactly the same iii) and 3D fluorescence quantification was not precise. However, the sum of intensity of all the pixels of entire images confirmed the two distinct profiles of nanobeads cellular-interactions and/or uptake. Our results demonstrated that PS nanobeads strongly interacted with Calu-3 cells during the first 4 h of exposure (Fig 3A), while PS nanobeads were continuously internalized within THP-1 macrophages during 24 h (Fig 3B). Furthermore, the mechanism of NPs uptake by Calu-3 cells would be interesting to clarify, as it was recently reported that PS nanobeads could induce changes in ion transport channels in this cell line [50]. In order to quantitatively follow these uptakes, we also performed flow cytometry analyses. Even though this approach requires cell trypsinization and detects NPs-cells interactions rather than effective NPs internalization, it allows a highly sensitive detection since it detects as few as 5–10 NPs per cell [52]. Flow cytometry showed that PS-NF and PS-COOH nanobeads were mainly in contact with Calu-3 cells until 4 h of exposure, followed by a strong decrease of nanobeads-cells interactions at 24 h (S5 Fig). Moreover, we confirmed the particular profile observed in confocal video-microscopy: PS-NH_2_ nanobeads were in contact around and/or within cells during the first two hours, and then nanobeads-cells interactions decreased until 4 h of exposure and increased again at 24 h (S5A Fig). This specific kinetic profile strongly suggested a transient exocytosis and/or efflux/discharge of PS-NH_2_ nanobeads from 2 to 4 h after exposure. It could be interesting to investigate the possible involvement of multi-drug resistance pumps in such phenomenon (using for example specific inhibitor such as verapamil and/or siRNA strategies). To precise the location of PS-NH_2_ nanobeads within and/or around Calu-3 cells, we also performed confocal fluorescence microscopy analysis. We confirmed that PS-NH_2_ nanobeads were mainly located around Calu-3 cell islets at 1 and 2 h, faintly detected at 4 h, and again detected at 24 h (Fig 4A). Similar experiments were performed on THP-1 macrophages and showed that PS-NH_2_ nanobeads were effectively internalized and mainly located in the cytoplasm rather than in the nuclei (Fig 4B and S4B Fig), corroborating data from the literature [11].

We then investigated the genotoxic effects of these nanobeads together with reduced GSH dosage, as genotoxicity and oxidative stress could be related [47,48]. The GSH dosage method was chosen rather than the ROS approach due to strong interferences observed between PS nanobeads fluorescence and CM-H_2_DCFDA or Mitosox probes (data not shown). Indeed, the excitation and emission wavelenghts of PS nanobeads and ROS probes are very close and prevents to accurate study measurements. In order to study genotoxicity we analyzed γ-H2Ax-foci, which is a very sensitive method to detect DNA double strand breaks [31] and recently described as a powerful method to predict *in vivo* genotoxicity [32]. As seen in Table 2, NPs functionalization strongly impacted genotoxicity, however with variation depending on the dose of PS nanobeads. PS-NF nanobeads did not significantly induce genotoxicity for concentrations up to 8.1 μg/cm^2^ in both cell lines. PS-COOH nanobeads were not genotoxic for Calu-3 cells (Table 2) but genotoxic for THP-1 macrophages (Table 2). These data should be interpreted together with PS nanobeads uptake since PS-COOH were detected into macrophages (S4B Fig) and faintly in Calu-3 cells (S4A Fig). However, we found that PS-NH_2_ nanobeads induced DNA double strand breaks (Table 2) while being either mainly around (Calu-3 cells, Fig 4A) or within the cells (THP-1 macrophages, Fig 4B). In addition, it was published that NPs were able to generate oxidative stress [47], which can lead to ROS generation and GSH depletion [53]. NH_2_ functionalization was shown to lead to the highest GSH depletion both on Calu-3 cells (S6C Fig) and THP-1 macrophages (S6F Fig). Interestingly, these results are in complete agreement with γ-H2Ax-foci results, since the highest number of DNA double strand breaks was observed after PS-NH_2_ nanobeads exposure (Table 2). Since we did not detect PS nanobeads in the nucleus in either cell line, we can suggest that genotoxicity may be related to a non-direct effect through ROS generation [54]. Primary indirect genotoxicity could be hypothesized as PS nanobeads exposure depleted anti-oxidants (S6 Fig), thus potentially increasing free radical levels that could cause DNA oxidative damages [55]. It could also be of interest to perform Calu-3 cells and THP-1 macrophages co-culture exposures that could better mimic the *in vivo* pulmonary barrier. Interestingly, it was showed that exposition of A549:THP-1 co-cultures to diesel exhaust NPs did not trigger significant oxidative DNA damage, compared to A549 epithelial cells in mono-cultures [54].

## Conclusion

In conclusion, our results clearly indicate that NPs surface chemistry is one of key features in nanotoxicology, conferring to NPs potential cytotoxic and/or genotoxic effects and that NPs induced genotoxicity is not directly related to NPs cellular uptake. Thus, NPs surface chemistry must be cautiously taken into account for NPs safer design, especially in nanomedicine issues.

## Methods

### Nanobeads

Orange fluorescent PS nanobeads (λ_ex_: 475 nm, λ_em_: 540 nm) were purchased from Magsphere Inc. The following nanobeads were tested: 51 nm non-functionalized (PS-NF) (Ref. PSOF050NM), 48 nm carboxylated (PS-COOH) (Ref. CAOF050NM) and 52 nm aminated (PS-NH_2_) (Ref. AMOF050NM). PS nanobeads were suspended at a concentration of 1 mg/mL in RPMI-1640 medium without phenol red supplemented with 5% (v/v) FBS and 1% (v/v) Penicillin-Streptomycin (Invitrogen) (called complete medium). PS-NF and PS-COOH nanobeads were indirectly sonicated 2 min (20 s pulses on/off) at 30 W with a cup-horn (Sonicator S-4000, Misonix Incorporated) at room temperature, while PS-NH_2_ nanobeads were just vortexed before use. Then, particle size distribution and zeta potential were measured using a Zetasizer Nano ZS (Malvern). A Scanning Transmission Electron Microscope (STEM) (STEM CM12, lab 6, electron gun 120 kV, Philips) was used to examine size and morphology of the nanobeads after dispersion. To analyze the chemical composition of the samples, an energy-dispersive X-ray microanalyzer equipped with a Super Ultra Thin Window (SUTW) model SAPPHIR (EDAX) was used.

### Cell culture

Human Calu-3 epithelial cells (ATCC number: HTB-55) and THP-1 monocytic cells (ATCC number: TIB-202) were routinely grown at 37°C in a humidified atmosphere of 5% CO_2_ and 95% air, in RPMI-1640 medium supplemented with 10% (v/v) FBS and 1% (v/v) Penicillin-Streptomycin (Invitrogen). THP-1 cells are non-adherent monocytic cells maintained between 10^5^ and 10^6^ cells/ml. Before their use in our study, they were differentiated in adherent macrophages by incubation with 50 nM of PMA (Phorbol 12-myristate 13-acetate) (Sigma-Aldrich, Ref. P1585, CAS Number 16561-29-8) during 24 h as previously described [35]. Medium used for nanobeads exposure was RPMI-1640 medium without phenol red supplemented with 5% (v/v) FBS and 1% (v/v) Penicillin-Streptomycin (Invitrogen) for both cell lines. For all experiments, Calu-3 cells were seeded at least 48 h before the beginning of exposure, while THP-1 cells were seeded and differentiated in adherent macrophages at least 24 h before the beginning of nanobeads exposure.

### Impedance measurements with the xCELLigence system

Impedance measurement is a dimensionless parameter termed Cell Index (CI) which is derived as a relative change in measured electrical impedance to represent cell status. Several features of CI are taken into account in the measure: i) when cells are not present or are not well-adhered on the electrodes, the CI is zero ii) under the same physiological conditions, when more cells are attached on the electrodes, the CI values are larger. Thus, CI is a quantitative measure of cell number present in a well iii) additionally, change in a cell status, such as cell morphology, cell adhesion, or cell viability will lead to a change in CI.

Background of the E-plates (specifics 96 wells microplates covered with electrodes) (ACEA Biosciences) was determined in 50 μL/well of medium and subsequently 150 μL of Calu-3 or THP-1 cell suspensions were added (1.5x10^4^ and 8x10^4^ cells per well, respectively). Then cells were grown for at least 24 h for THP-1 and 48 h for Calu-3, with impedance measured every 5 min during 6 h, then every 15 min until addition of nanobeads. Cells were exposed to PS nanobeads at concentrations from 0.3, 1.6, 8.1, 16.1 and 32.3 μg/cm^2^. Signal was monitored every 5 min during 6 h (early effects), then every 10 min until the end of experiment (late effects). Cell index (CI) raw data values were calculated as follows: Zi-Z0 [Ohm]/15[Ohm]; where Z0: is the background resistance and Zi: the individual time point resistance). Normalized cell index was also calculated by the software at the selected normalization time point, which was chosen as time just before the addition of nanoparticles in order to minimize inter-wells variability before the beginning of exposure.

### alamarBlue viability assay

Calu-3 cells (4x10^4^ cells/well) and THP-1 differentiated macrophages (8x10^4^ cells/well) were exposed to PS-NF, PS-COOH and PS-NH_2_ nanobeads at same range of concentrations detailed above. Cell viability was evaluated according with the Invitrogen alamarBlue protocol [56–58].

Cells were washed two times with HBSS containing CaCl_2_ and MgCl_2_. Then 100 μL of a mix containing RPMI-1640 medium (without phenol red) supplemented with 5% (v/v) FBS, 1% (v/v) Penicillin-Streptomycin (Invitrogen) plus 10% (v/v) alamarBlue were added in each well. After 3 h of incubation at 37°C in a humidified atmosphere of 5% CO_2_ and 95% air, fluorescence was measured with a cyto-fluorometer adapted to micro-plate (λ_ex_: 555 nm, λ_em_: 585 nm) (TECAN Infinite M2000, Switzerland).

### Video-microscopy

For each experiment, 2.5x10^5^ Calu-3 cells 10^6^ THP-1 macrophages were platted on uncoated 12-well glass bottom dishes 14 mm (MatTek). Fluorescent images were captured through a Plan Apo 40x DIC objective (NA: 0.95) on a Nikon A1R confocal laser scanning microscope system attached to an inverted ECLIPSE Ti (Nikon Corp., Tokyo, Japan) thermostated at 37°C under 5% of CO_2_ atmosphere. All PS nanobeads were excited at 488 nm. The fluorescence emission wavelengths were collected between 550 nm and 590 nm for each PS nanobeads. Images were acquired at 512x512 pixels format with a 640 nm/pixel resolution. Images captions were performed every 15 min during 6 h, then every 30 min until 24 h. Then, a representative field of each caption set was cut off using Adobe Photoshop CS5 (San Jose, CA).

### Flow cytometry for study of cells-PS nanobeads interactions

To investigate interactions between PS nanobeads and Calu-3 cells and THP-1 macrophages, exposures to 8.1 μg/cm^2^ for each PS nanobeads were performed in 6-well dishes during 1, 2, 4 and 24 h. Then Calu-3 cells were trypsinized while THP-1 macrophages were harvested using Cell Dissociation Buffer, Enzyme Free, Hanks'-Based (Invitrogen, Ref. 13150–016). Cells were then centrifugated 5 min at 150xg and the pellet was resuspended in DPBS 1X (containing CaCl_2_ and MgCl_2_) in flow cytometry compatible tubes. Data were collected on BD Facscalibur (Becton, Dickinson and Company; Franklin Lakes, NJ) and PS nanobeads fluorescence was collected on FL2 channel. Then all data were analysed by using FlowJo 7.5.5 software (Tree Star Inc.) (Ashland, OR).

### PS nanobeads uptake using confocal microscopy

Confocal microscopy was used to precise nanobeads location within and/or around exposed cells. As PS nanobeads are fluorescent, we decided to label nuclei with Hoechst 33342 and to stain actin with Alexa Fluor 635 Phalloidin. For each experiment, 8x10^4^ cells/well for Calu-3 cells and 1.8x10^5^ THP-1 macrophages/well were seeded on Lab-Tek II Chamber Slide 8 wells (Nunc). Then cells were exposed to 8.1 μg/cm^2^ of nanobeads during 1, 2, 4 and 24 h. After treatment, cells were washed twice with 200 μL of PBS 1X and fixed for 15 min with PFA 4%, washed twice with 200 μL of PBS 1X and then permeabilized for 10 min at room temperature in [PBS 1X and Triton 0.1%]. Cells were stained with a mix of 200 μL/well of [1 μg/mL of Hoechst 33342 + 5 μL Alexa Fluor 635 Phalloidin in water] during 20 min at 37°C. Samples were washed with 3×300 μL of [PBS 1X, Triton 0.025%] before mounting in ProLong Gold antifade reagent (Invitrogen) in order to proceed for confocal microscopy acquisitions. Fixed and labeled cells were photographed with same equipment as the one used for γ-H2Ax foci counts. Even though all chosen dyes do not present overlap between the emission wavelengths, we have ensured that we did not detect signal overlap for each single set. Each analysis was made from 3 independents triplicates. Confocal microscopy optical slice sections of 10 to 20 μm were made from the luminal to the basal pole of the cells, each acquisition containing at least 30 stacks. Images were prepared by using Adobe Photoshop CS5 (San Jose, CA) software for overlay generation.

### γ-H2Ax-foci

Confocal microscopy was used to quantitatively investigate NPs induced genotoxicity by the detection of γ-H2Ax-foci, which is a very sensitive method to detect DNA double strand breaks [31]. As PS 50 nm nanobeads are fluorescent we decided to label nuclei with Hoechst 33342 and to secondary detect γ -H2Ax-foci with Alexa Fluor 488. For each experiment, 1.8x10^5^ THP-1 macrophages per well were seeded on Lab-Tek II Chamber Slide 8 wells (Nunc) 24 h before exposure while 8x10^4^ Calu-3 cells were seeded on same culture support at least 48 h before exposure. Then cells were exposed to PS nanobeads during 1, 2, 4 and 24 h at 0.3 and 8.1 μg/cm^2^. After treatment, cells were washed twice with 200 μL of 1X PBS and fixed for 15 min with PFA 4%, washed twice with 200 μL of 1X PBS and then permeabilized for 10 min at room temperature in [1X PBS and Triton 0.1%]. Cells were blocked in [1X PBS, 0.025%, 10% of goat serum (Jackson ImmunoResearch)] for 1 h at room temperature, then incubated for 75 min at room temperature with 1:500 of monoclonal γ-H2Ax antibody (Anti-phospho-Histone H2Ax (Ser139), clone JBW301, Ref. 05–636, Upstate Millipore), washed with 3×300 μL of [1X PBS, Triton 0.025%] and then incubated at room temperature for 60 min with 1:500 dilution of Alexa Fluor 488 goat anti-mouse IgG (H+L) (Invitrogen) as a secondary antibody. Samples were washed with 3×300 μL of [1X PBS, Triton 0.025%] and cells were incubated at 37°C for 20 min with 200 μL of Hoechst 33342 (Invitrogen) at 1 μg/mL in water. Finally, Lab-Tek Chamber Slide 8 wells were washed with 3×300 μL of [1X PBS, Triton 0.025%] before mounting in ProLong Gold antifade reagent (Invitrogen) in order to proceed for confocal microscopy visualization.

### **Confocal microscopy and** γ**-H2Ax foci counts**

Fixed and labeled cells were photographed with an ACS APO 40X oil CS (NA 1.15) objective under a fluorescence confocal microscope (Leica TCS SPE, Wetzlar, Germany) equipped with 4 diode lasers (405, 488, 532 and 635 nm). All the settings and proceedings of the acquisitions were already extensively described in one of our previous studies [59]. Acquisitions were performed on at least 200 nuclei and 10 fields. The spectral sliders were set in sequential mode and by decreasing excitation wavelengths, to maximize signal and to reduce spectral overlap. Each experiment was performed using same acquisition settings for all nanobeads (laser intensity, objectives, etc.). All details concerning proceedings of the acquisitions are exactly the same as the ones used in one of our previous study [59]. Even though all chosen dyes do not present overlap between the emission wavelengths, we have ensured that we did not detect signal overlap for each single set. Each analysis was made from 3 independents triplicates on at least 200 cells and at least 10 images of each condition were analyzed. Confocal microscopy optical slice sections of 10 to 20 μm were made from the apical to the basal pole of the cells, each acquisition containing 9 stacks. Images were prepared and stacked with ImageJ software [60] by using the stacks tool. Then the tiff images were converted into 8 bits before performing foci counts. Cell Profiler software [61] was used for the detection and scoring of foci in Alexa Fluor images.

### Intracellular reduced GSH assay

As previously described in literature [62,63], we performed a monochlorobimane (mBCI) assay (Life Technologies). This probe is essentially non fluorescent until conjugated and readily reacts with reduced GSH through GSH transferase to form a fluorescent derivative. A 100 μL mix containing 100 μM of mBCI in HBSS (with CaCl_2_ and MgCl_2_) was directly added in each well to nanobeads treated cells for 15 min before fluorometric detection with a cyto-fluorometer adapted to micro-plate (λ_ex_: 360 nm, λ_em_: 480 nm) (TECAN Infinite M2000, Switzerland).

### Statistical Analysis

All data are represented as mean ±SD of three independent experiments. Data of GSH and alamarBlue assays were analyzed by one-way analysis of variance (ANOVA) followed by Dunnett’s t-test to compare the different treated groups to the control (α risk = 0.05), using GraphPad Instat 3 software (La Jolla, CA). To test whether the basal yield of γ-H2Ax foci observed in control cells was significantly different from that observed in exposed-cells for both of the three tested PS nanobeads, a Wilcoxon rank test based on at least 200 observations for each condition was performed using R software [64].

## Supporting Information

S1 Fig PS-NH_2_ nanobeads characterization (complementary results). Zeta potentials and PDI of PS-NH_2_ nanobeads suspended in RPMI 1640 supplemented with 5% (v/v) FBS (A). Data represent the mean ± SD of three independent experiments. Measurements were performed on simply vortexed sample (blue curve) and after sample sonication using cup horn probe (green curve). Cup-horn sonication induces a polydispersity of the sample while a simple vortex preserves the monodispersity of the sample.
(DOCX)

S2 Fig Cell index real-time monitoring of THP-1 monocytes (red curve) and THP-1 differentiated macrophages (black curve). Differentiation was obtained after 24 h of incubation to 50 nM of PMA. Impedance measurements were carried out for 50 h and cell indexes (one representative experiment among three independents experiments) were normalized at time 0 to ensure non inter-wells variability. CI values were layered to the background signal with CI values near to 0 during the whole experiment due the absence of cell adherence for THP-1 monocytes.
(DOCX)

S3 Fig Calu-3 and THP-1 cells viability estimated by alamarBue assay. Cell viability was measured for Calu-3 (column 1) and THP-1 cells (column 2) after 2, 4, 24 and 48 h of exposure to the three kinds of PS nanobeads. Data represent the mean percentage of control ± SD of three independent experiments. One-way ANOVA and Dunett post-test (comparisons *versus* control cells not exposed to NPs) were performed (* *p*<0.05; ** *p*<0.01).
(DOCX)

S4 Fig Video-microscopy captures of Calu-3 cells (A) and THP-1 macrophages (B) exposed to PS nanobeads. Representative fields of Calu-3 control cells (A, column 1) and exposed cells to PS-NF (A, column 2), PS-COOH (A, column 3) and PS-NH_2_ (A, column 4) nanobeads at t_0_ (line1), 1 (line 2), 2 (line 3), 4 (line 4) and 24 h (line 5). Representative fields of control THP-1 macrophages (B, column 1) and exposed cells to PS-NF (B, column 2), PS-COOH (B, column 3) and PS-NH_2_ (B, column 4) nanobeads at t_0_ (line1), 1 (line 2), 2 (line 3), 4 (line 4) and 24 h (line 5). Each representative field comes off video microscopy captions. White bars correspond to 10 nm and white arrows indicate fluorescent nanobeads.
(DOCX)

S5 Fig Cells-PS nanobeads interactions measured by flow cytometry for Calu-3 cells (A) and THP-1 macrophages (B). Percentages of positive and negative cells (Nano+ and Nano-, respectively) were determined by nanobeads fluorescence emission. Data represent the mean percentage ± SD of three independent experiments.
(DOCX)

S6 Fig GSH gluthatione depletion dosages obtained for Calu-3 cells and THP-1 macrophages exposed to PS nanobeads. Intracellular reduced GSH evaluation after exposure of Calu-3 (column 1) cells and THP-1 macrophages (column 2) to PS nanobeads. Cells were exposed for 1, 2, 4 or 24 h to PS-NF (A and D), PS-COOH (B and E) or PS-NH_2_ (C and F) nanobeads. GSH level was evaluated with mBCI fluorogen probe and data represent the mean percentage of control ± SD of three independent experiments. One-way ANOVA and Dunett post-test (comparisons *versus* control cells not exposed to PS nanobeads) were performed (* *p*<0.05; ** *p*<0.01).
(DOCX)
