# Supplementary material for: Specific Uptake and Genotoxicity Induced by Polystyrene Nanobeads with Distinct Surface Chemistry on Human Lung Epithelial Cells and Macrophages
Source: PLoS One. 2015 Apr 15;10(4):e0123297. doi: 10.1371/journal.pone.0123297 (PMC4398494; doi:10.1371/journal.pone.0123297)
Supplement: S1 Fig — Zeta potentials and PDI of PS-NH2 nanobeads suspended in RPMI 1640 supplemented with 5% (v/v) FBS (A). Data represent the mean ± SD of three independent experiments. Measurements were performed on simply vortexed sample (blue curve) and after sample sonication using cup horn probe (green curve). Cup-horn sonication induces a polydispersity of the sample while a simple vortex preserves the monodispersity of the sample. (DOCX) [file pone.0123297.s001.docx]

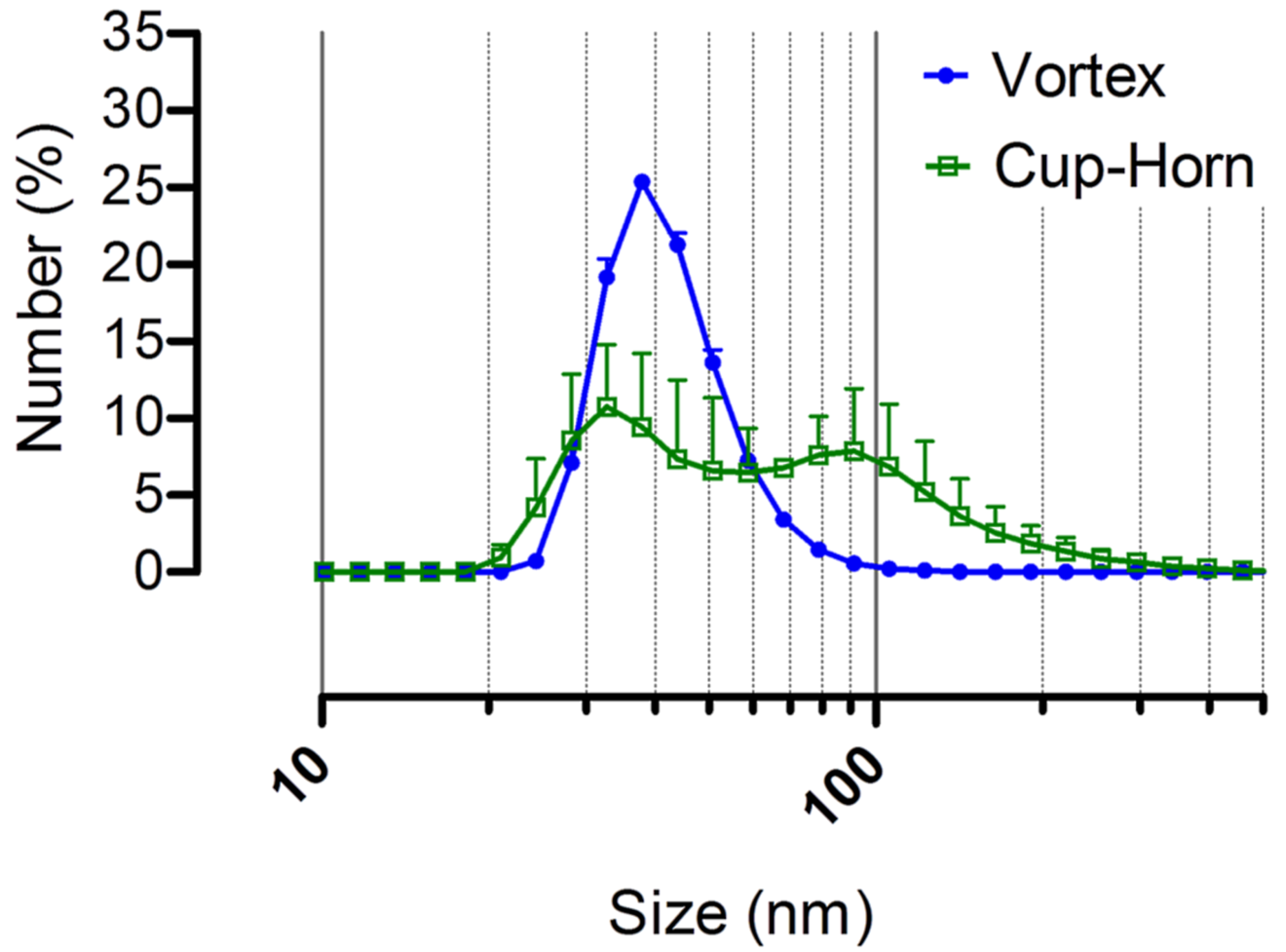


**S1 Fig.** **PS-NH_2_ nanobeads characterization (complementary results).** Zeta potentials and PDI of PS-NH_2_ nanobeads suspended in RPMI 1640 supplemented with 5% (v/v) FBS (A). Data represent the mean ± SD of three independent experiments. Measurements were performed on simply vortexed sample (blue curve) and after sample sonication using cup horn probe (green curve). Cup-horn sonication induces a polydispersity of the sample while a simple vortex preserves the monodispersity of the sample.
