# Supplementary material for: Specific Uptake and Genotoxicity Induced by Polystyrene Nanobeads with Distinct Surface Chemistry on Human Lung Epithelial Cells and Macrophages
Source: PLoS One. 2015 Apr 15;10(4):e0123297. doi: 10.1371/journal.pone.0123297 (PMC4398494; doi:10.1371/journal.pone.0123297)
Supplement: S2 Fig — Differentiation was obtained after 24 h of incubation to 50 nM of PMA. Impedance measurements were carried out for 50 h and cell indexes (one representative experiment among three independents experiments) were normalized at time 0 to ensure non inter-wells variability. CI values were layered to the background signal with CI values near to 0 during the whole experiment due the absence of cell adherence for THP-1 monocytes. (DOCX) [file pone.0123297.s002.docx]

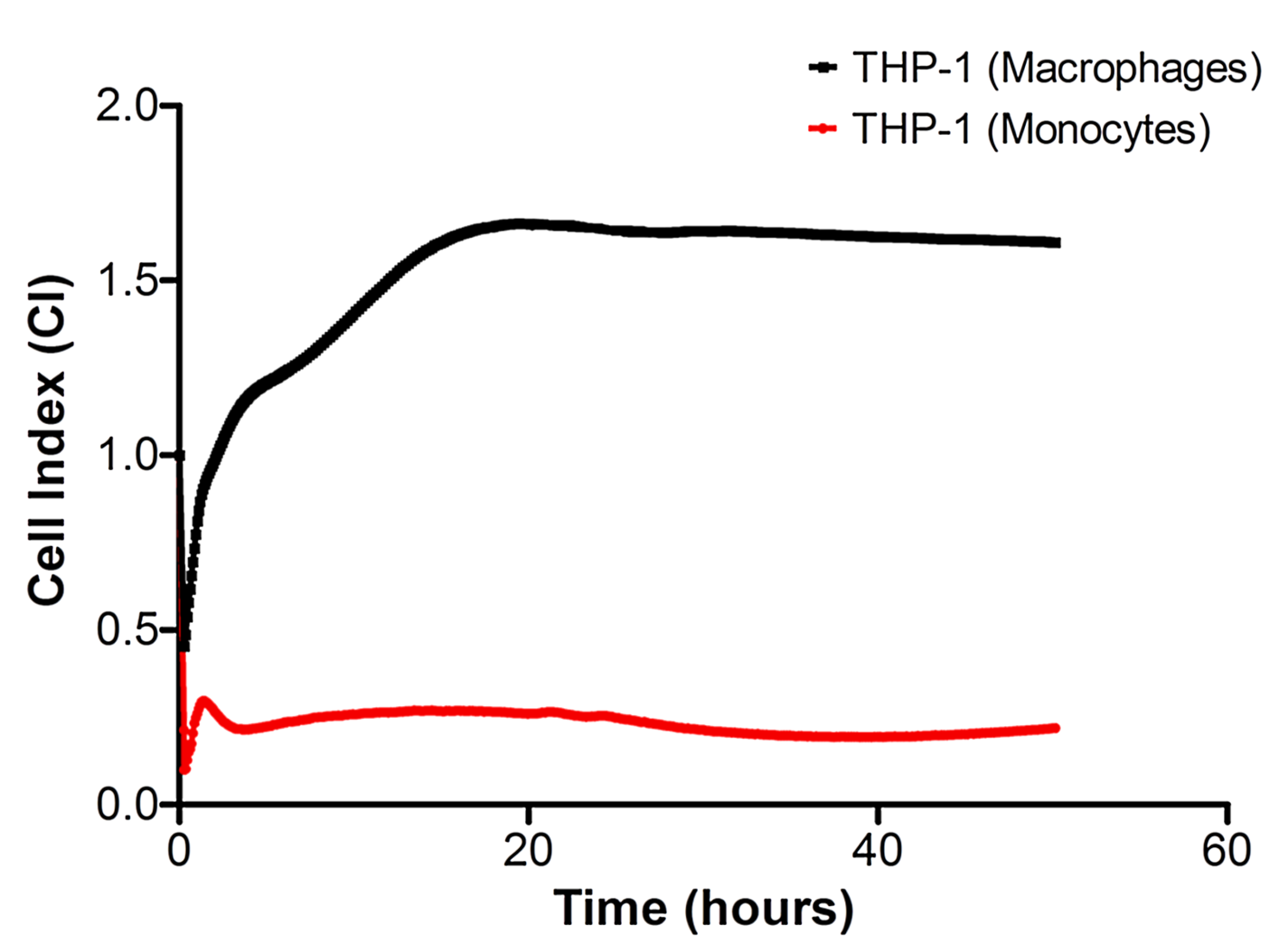


**S2 Fig.** **Cell index real-time monitoring of THP-1 monocytes (red curve) and THP-1 differentiated macrophages (black curve).** Differentiation was obtained after 24 h of incubation to 50 nM of PMA. Impedance measurements were carried out for 50 h and cell indexes (one representative experiment among three independents experiments) were normalized at time 0 to ensure non inter-wells variability. CI values were layered to the background signal with CI values near to 0 during the whole experiment due the absence of cell adherence for THP-1 monocytes.
