# Supplementary material for: Specific Uptake and Genotoxicity Induced by Polystyrene Nanobeads with Distinct Surface Chemistry on Human Lung Epithelial Cells and Macrophages
Source: PLoS One. 2015 Apr 15;10(4):e0123297. doi: 10.1371/journal.pone.0123297 (PMC4398494; doi:10.1371/journal.pone.0123297)
Supplement: S3 Fig — Cell viability was measured for Calu-3 (column 1) and THP-1 cells (column 2) after 2, 4, 24 and 48 h of exposure to the three kinds of PS nanobeads. Data represent the mean percentage of control ± SD of three independent experiments. One-way ANOVA and Dunett post-test (comparisons versus control cells not exposed to NPs) were performed (* p<0.05; ** p<0.01). (DOCX) [file pone.0123297.s003.docx]

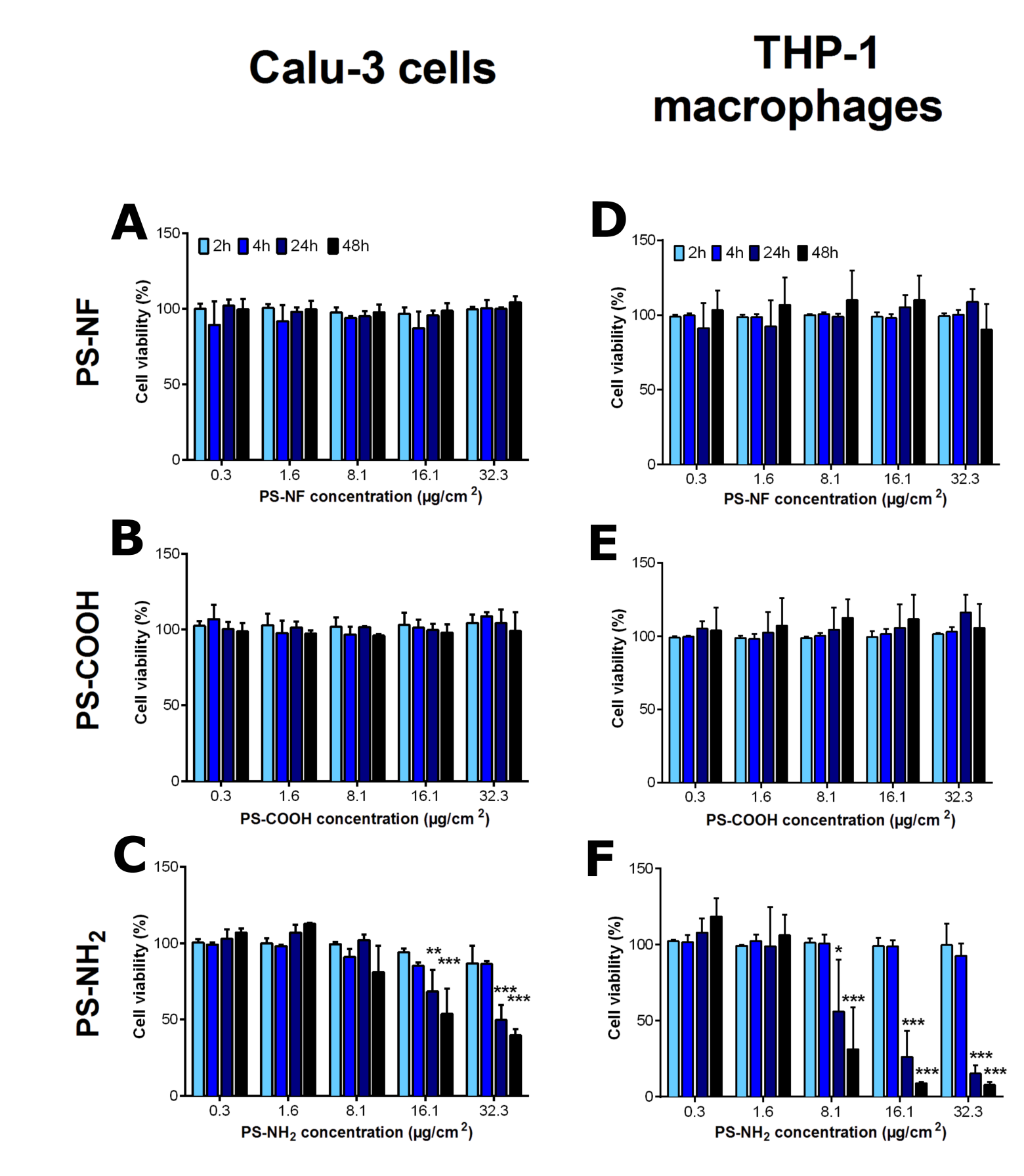


**S3 Fig. Calu-3 and THP-1 cells viability estimated by alamarBue® assay.** Cell viability was measured for Calu-3 (column 1) and THP-1 cells (column 2) after 2, 4, 24 and 48 h of exposure to the three kinds of PS nanobeads. Data represent the mean percentage of control ± SD of three independent experiments. One-way ANOVA and Dunett post-test (comparisons *versus* control cells not exposed to NPs) were performed (* *p*<0.05; ** *p*<0.01).
