# Supplementary material for: Specific Uptake and Genotoxicity Induced by Polystyrene Nanobeads with Distinct Surface Chemistry on Human Lung Epithelial Cells and Macrophages
Source: PLoS One. 2015 Apr 15;10(4):e0123297. doi: 10.1371/journal.pone.0123297 (PMC4398494; doi:10.1371/journal.pone.0123297)
Supplement: S4 Fig — Representative fields of Calu-3 control cells (A, column 1) and exposed cells to PS-NF (A, column 2), PS-COOH (A, column 3) and PS-NH2 (A, column 4) nanobeads at t0 (line1), 1 (line 2), 2 (line 3), 4 (line 4) and 24 h (line 5). Representative fields of control THP-1 macrophages (B, column 1) and exposed cells to PS-NF (B, column 2), PS-COOH (B, column 3) and PS-NH2 (B, column 4) nanobeads at t0 (line1), 1 (line 2), 2 (line 3), 4 (line 4) and 24 h (line 5). Each representative field comes off video microscopy captions. White bars correspond to 10 nm and white arrows indicate fluorescent nanobeads. (DOCX) [file pone.0123297.s004.docx]

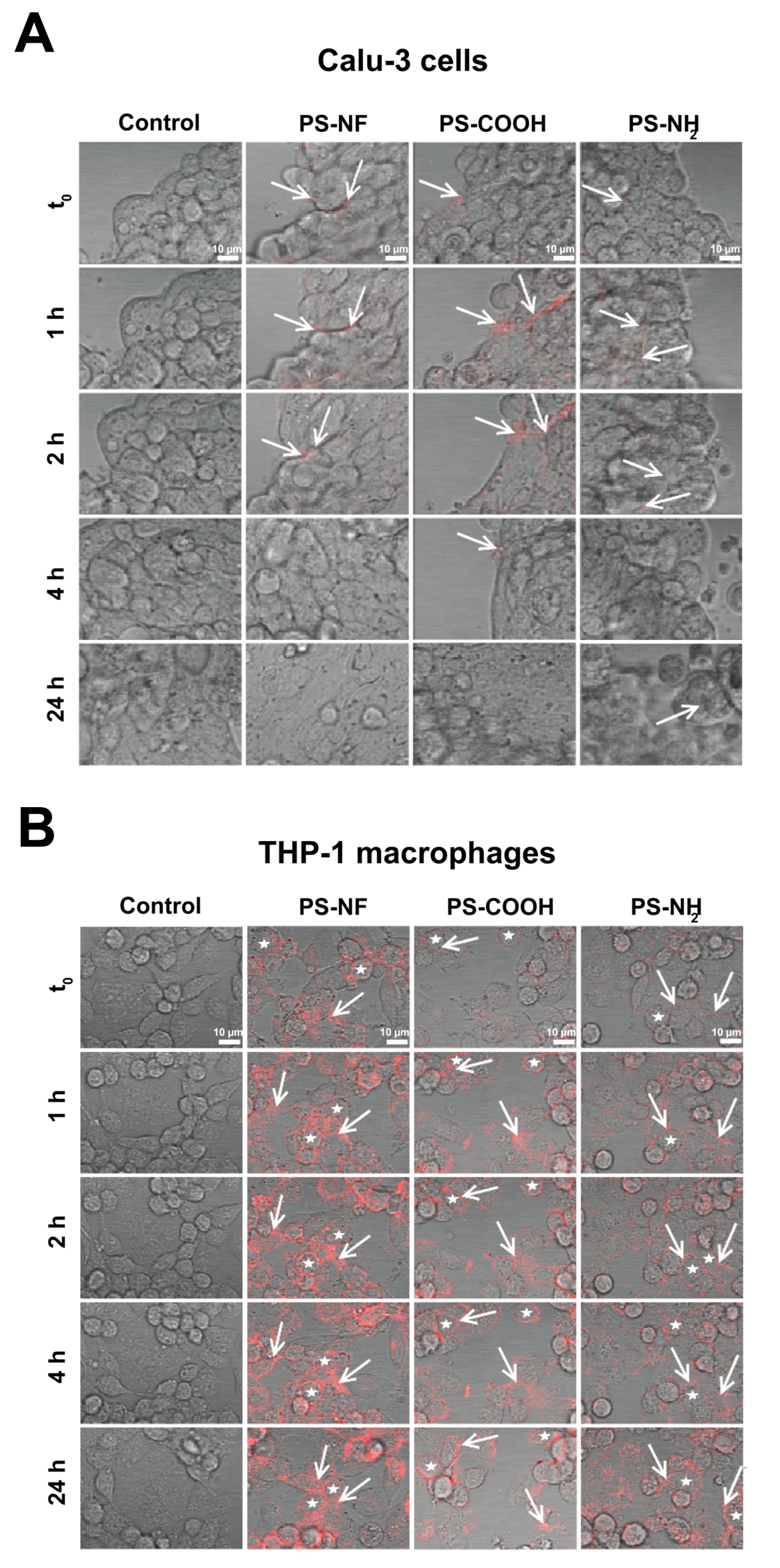


**S4 Fig. Video-microscopy captures of Calu-3 cells (A) and THP-1 macrophages (B) exposed to PS nanobeads.** Representative fields of Calu-3 control cells (A, column 1) and exposed cells to PS-NF (A, column 2), PS-COOH (A, column 3) and PS-NH_2_ (A, column 4) nanobeads at t_0_ (line1), 1 (line 2), 2 (line 3), 4 (line 4) and 24 h (line 5). Representative fields of control THP-1 macrophages (B, column 1) and exposed cells to PS-NF (B, column 2), PS-COOH (B, column 3) and PS-NH_2_ (B, column 4) nanobeads at t_0_ (line1), 1 (line 2), 2 (line 3), 4 (line 4) and 24 h (line 5). Each representative field comes off video microscopy captions. White bars correspond to 10 nm and white arrows indicate fluorescent nanobeads.
