# Supplementary material for: Specific Uptake and Genotoxicity Induced by Polystyrene Nanobeads with Distinct Surface Chemistry on Human Lung Epithelial Cells and Macrophages
Source: PLoS One. 2015 Apr 15;10(4):e0123297. doi: 10.1371/journal.pone.0123297 (PMC4398494; doi:10.1371/journal.pone.0123297)
Supplement: S5 Fig — Percentages of positive and negative cells (Nano+ and Nano-, respectively) were determined by nanobeads fluorescence emission. Data represent the mean percentage ± SD of three independent experiments. (DOCX) [file pone.0123297.s005.docx]

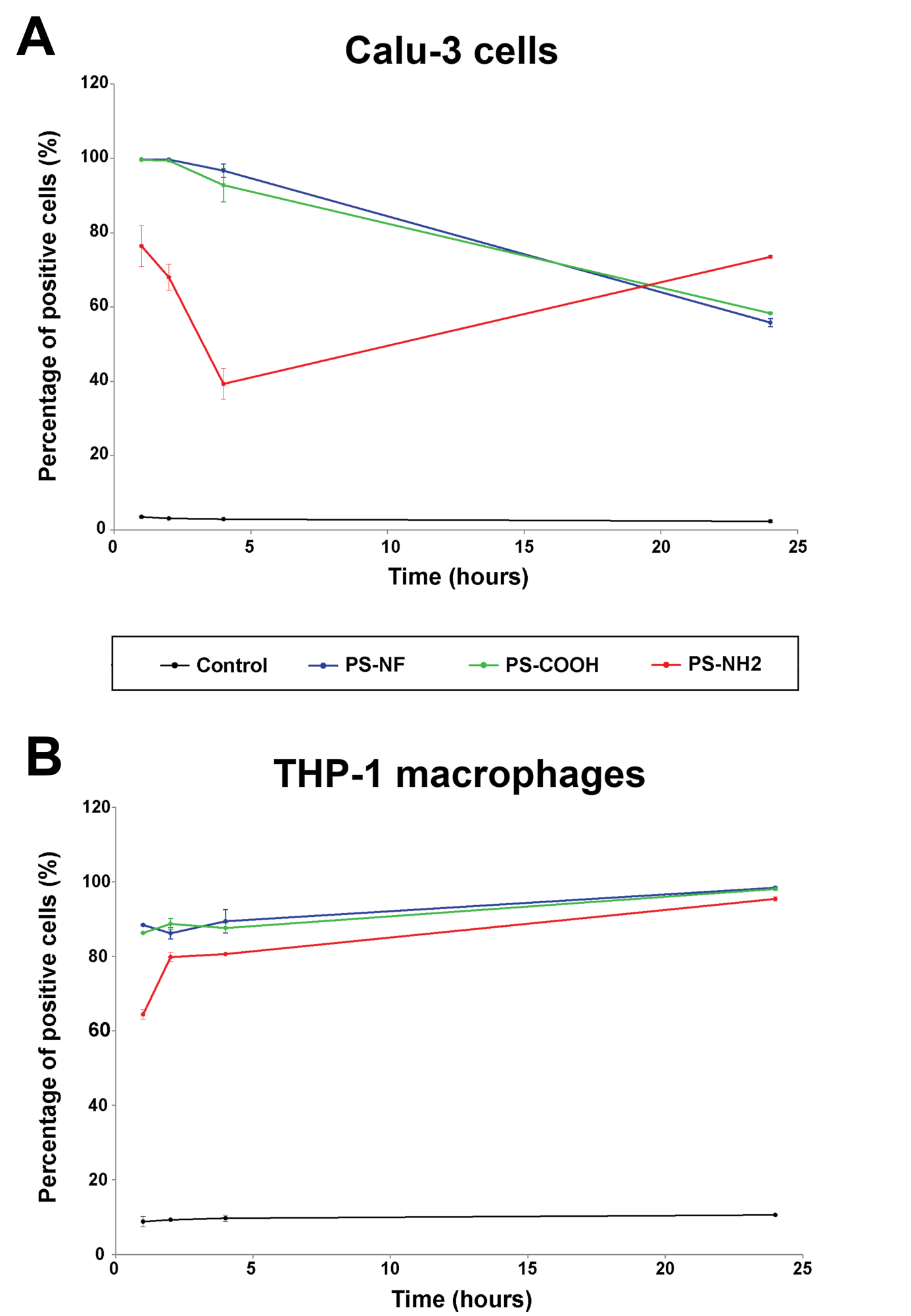


**S5 Fig.** **Cells-PS nanobeads interactions measured by flow cytometry for Calu-3 cells (A) and THP-1 macrophages (B).** Percentages of positive and negative cells (Nano+ and Nano-, respectively) were determined by nanobeads fluorescence emission. Data represent the mean percentage ± SD of three independent experiments.
