# Supplementary material for: Specific Uptake and Genotoxicity Induced by Polystyrene Nanobeads with Distinct Surface Chemistry on Human Lung Epithelial Cells and Macrophages
Source: PLoS One. 2015 Apr 15;10(4):e0123297. doi: 10.1371/journal.pone.0123297 (PMC4398494; doi:10.1371/journal.pone.0123297)
Supplement: S6 Fig — Intracellular reduced GSH evaluation after exposure of Calu-3 (column 1) cells and THP-1 macrophages (column 2) to PS nanobeads. Cells were exposed for 1, 2, 4 or 24 h to PS-NF (A and D), PS-COOH (B and E) or PS-NH2 (C and F) nanobeads. GSH level was evaluated with mBCI fluorogen probe and data represent the mean percentage of control ± SD of three independent experiments. One-way ANOVA and Dunett post-test (comparisons versus control cells not exposed to PS nanobeads) were performed (* p<0.05; ** p<0.01). (DOCX) [file pone.0123297.s006.docx]

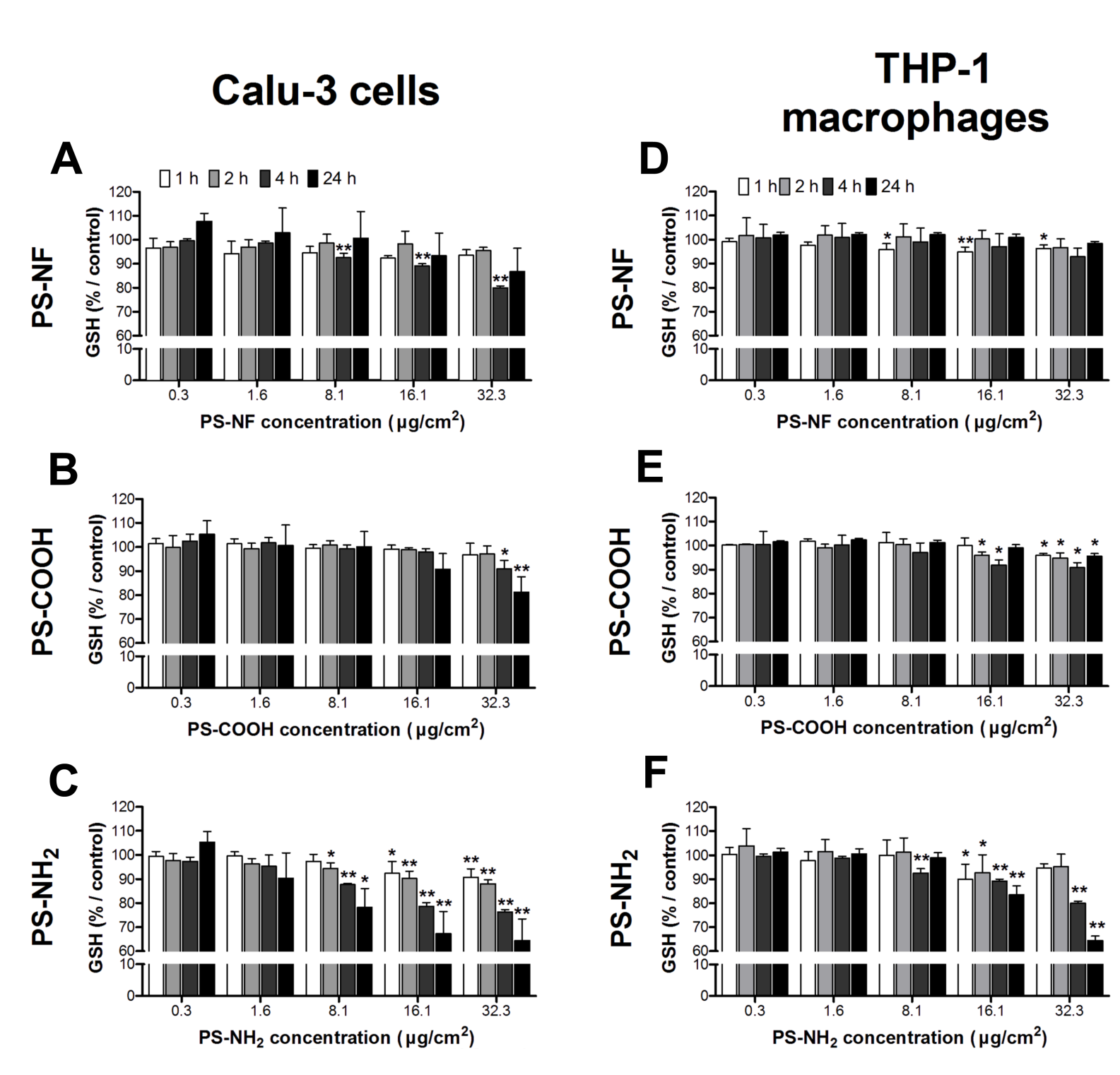


**S6 Fig. GSH gluthatione depletion dosages obtained for Calu-3 cells and THP-1 macrophages exposed to PS nanobeads.** Intracellular reduced GSH evaluation after exposure of Calu-3 (column 1) cells and THP-1 macrophages (column 2) to PS nanobeads. Cells were exposed for 1, 2, 4 or 24 h to PS-NF (A and D), PS-COOH (B and E) or PS-NH_2_ (C and F) nanobeads. GSH level was evaluated with mBCI fluorogen probe and data represent the mean percentage of control ± SD of three independent experiments. One-way ANOVA and Dunett post-test (comparisons *versus* control cells not exposed to PS nanobeads) were performed (* *p*<0.05; ** *p*<0.01).
